# Association of risk behaviours, socio-economic characteristics and academic progress in adolescents: an analysis of the 1993 birth cohort in Pelotas, Brazil

**DOI:** 10.1080/02673843.2018.1564932

**Published:** 2019-01-11

**Authors:** Lívia Madeira Triaca, Andréa Dâmaso Bertoldi, Paulo de Andrade Jacinto, Helen Gonçalves, Ana Maria Baptista Menezes, Aluísio J. D. Barros, César Augusto Oviedo Tejada

**Affiliations:** a Department of Economics, Federal University of Rio Grande Foundation (Fundação Universidade Federal do Rio Grande – FURG), Rio Grande, Brazil; b Postgraduate Program in Epidemiology, Federal University of Pelotas (Universidade Federal de Pelotas - UFPel), Rio Grande do Sul, Brazil; c Department of Economics, Federal University of Paraná (Universidade Federal do Paraná – UFPR), Paraná, Brazil

**Keywords:** Education, health behavior, adolescence

## Abstract

To analyze how socio-economic factors and behavioural characteristics are related to the failure of academic progress. Data of the 1993 Birth Cohort of the city of Pelotas, Brazil, were analyzed using four follow-up waves. As a measure of the failure of academic progress, we used indicators of the age-grade gap. We analyzed the association of demographic, socio-economic, and behavioural characteristics. Factors associated with failure of academic progress were assessed through logistic regression. There are a higher odds of the age-grade gap when the adolescent is not white, man, of low socio-economic status, whose parents have low schooling and living in large families. In relation to risk behaviours, alcohol and tobacco consumption represent higher odds of the age-grade gap at age 18. The results show that socio-economic factors and behavioural characteristics are important predictors of academic progress. Public policies that seek to promote education should be targeted at the most vulnerable groups, decreasing the observed inequalities.

## Introduction

Education is a major concern in low- and middle-income countries (LMICS) as it is strongly related to economic growth and social development (OECD, 2012). The economic and social costs of the failure of academic progress are high, and the proper completion of high school imbue individuals with a better perspective of life through improved health and better jobs (OECD, 2012).

Many are the factors related to school success. Child and youth health is seen as a fundamental political issue for LMICS since investment in health, mainly during the first period of infancy, affects the potential development of the individual (Glewwe, 2005; Glewwe, Jacoby, & King, 2001). A series of empirical studies have shown evidence of the existence of a positive effect of health on education, reporting strong evidence that an improvement of health and nutrition promotes better educational outcomes (Alderman, Behrman, Lavy, & Menon, 2001; Glewwe & Jacoby, 1995; Glewwe et al., 2001; Gomes-Neto, Hanushek, Leite, & Frota-Bezzera, 1997).

Most studies have focused on measures of health in infancy and their impact on the development of the individual (Glewwe et al., 2001; Gomes-Neto et al., 1997). However, it is during adolescence that major transitions take place, with puberty and the rapid maturing of the brain leading to new behaviours and capabilities, causing transformations in various spheres – family, friends, education, and health (Viner et al., 2012). Some risk behaviours may be adopted during this period of the life cycle that might jeopardize educational performance and a person’s perspectives in adult life (Viner et al., 2012).

Risky behaviours adopted during adolescence may also threaten individual achievement. The evidence suggests that consumption of licit and illicit substances is associated with poor educational outcomes, such as lower educational aspirations, greater likelihood of dropping out, and poor academic performance (Horwood et al., 2010; Jeynes, 2002). However, demographic and socio-economic factors can also be associated with educational results. Students with a low socio-economic level and children of less educated parents have greater odds of achieving low academic performance (Hanushek, 1997; Woessmann, 2001). Socio-economic and risky behaviours can become obstacles for school success.

A better understanding of these relationships is of great importance for improving the living standards of the population. The return in investments made in the early years of life are higher than investments made in later periods; specific investments in this phase can be considered as important predictors of future productivity and economic success. Therefore, studies in this area would help to identify where government interventions are most adequate, contributing in a significant way for the formulation of public policies aimed at improving the quality of life. Based on longitudinal data from the 1993 birth cohort in Pelotas, the present study seeks to determine if socio-economic factors and behavioural characteristics are related to school performance. The analysis has been made in adolescents, aged 18, and the factors possibly associated were collected through four follow-ups.

## Method

### Participants and procedure

The city of Pelotas is located in the southernmost area of Brazil and has a population of around 330,000 inhabitants. Its economy is based on commerce and agribusiness and in spite of being situated in one of the most developed regions of the country, Pelotas presents a high concentration of income, with around 60% of its income on the hands of the wealthiest 20%, and according to municipal human development index (MHDI), Pelotas presents an educational index of 0.632, considered intermediate (the indexes of MHDI are expressed as a value between 0 and 1, 1 indicates a high level of development, 0 a very low level) (UNDP, 2013). This study is based on the 1993 Pelotas birth cohort, a longitudinal study that includes all live births in 1993 in the urban area of Pelotas (n = 5,249). Follow-ups were conducted at birth and at ages of 11, 15 and 18. Data regarding socio-economic and behavioural conditions were collected through interviews with the participant and the legal guardian. Methodological details of the 1993 birth cohort are available in other publications (Gonçalves et al., 2014; Victora et al., 2006).

All phases of the 1993 birth cohort of the city of Pelotas were approved by the Ethics Research Committee. A written Term of Informed Consent was signed by the mother or adult responsible in every visit, and verbal consent was given by the adolescents in the follow-up visits at the ages of 11 and 15. At the age of 18, the cohort members signed the consent term.

### Follow-ups

Four follow-up waves were used in this analysis. In 1993, mothers were interviewed within 24-h of giving birth and socio-economic data was collected. Follow-ups were carried out in 2004, 2008, and 2011, when the participants were, on average, aged 11, 15, and 18. Follow-up rates were, respectively, 87.5%, 85.7% and 81.3%.

## Measures

### Academic progress

Basic education in Brazil, when the 1993’s cohort started, comprised eight years of fundamental school and three years of intermediate school, starting at the age of seven (Law n. 9.394, 1996). As a measure of school performance, we used indicators of the age-grade gap that were built for the 2011 follow-up. Of the 5,249 cohort members, we registered the schooling of 3,852 participants in 2011. Since the participants turned 18 years old in 2011, the elaboration of the variable took into account only the academic level attained, and as a result, the participants that were in the third grade of intermediate school or who had already finished high school were considered adequate, while the adolescents in lower grades were considered lagging.

### Socio-economic and demographic characteristics

Parents’ education at birth was divided into four categories (0 to 4, 5 to 8, 9 to 11; and ≥12 years of schooling). The size of the family at the age of 11 was divided into three categories (2 to 3, 4 to 5, ≥6 members). To measure the socio-economic level an asset index was built. The index is based on principal components analysis and considers ownership of material goods and characteristics of the dwelling place. That method allows for the elaboration of a measure of the families’ long-term wealth (Filmer & Pritchett, 1998). We registered the socio-economic level of the participant during the 2004 period, divided into quintiles.

The work variable was included as a binary variable, which refers to work outside the home. Three work variables were created, for 2004, 2008, and 2008 follow-ups. Skin colour was classified into two groups, white and non-white, including black, brown (mixed), yellow (oriental) and indigenous.

### Risky behaviours

Participants’ consumption of alcohol and tobacco was recorded at the ages of 11, 15, and 18, representing the beginning, middle and end of adolescence. For all follow-ups were created variables that represent the use of the substance in the period (alcohol = 1 if the participant reported that drank more than once a month; and tobacco = 1 if the adolescent smokes at least once a week). After employing the test suggested by Mishra et al. (2008), we redefine the variables of tobacco and alcohol following the test results. The smoking variable shall refer to age adolescents began smoking and was divided into four categories (never, ≤12, 13 to 15, and >15 years). For alcohol, we included two dummy variables, one for the mobility of 11 to 15 years and another for 15 to 18. These variables represent the change in the status of consumption (1 = changed the status of consumption; 0 = if the consumption status was maintained in the period).

### Data analysis

Data were analyzed using the Stata software, version 12.0 (Stata Corp, College Station, TX, 2011). The factors associated with the failure of academic progress were assessed through the crude and adjusted analysis. The analyses were performed by means of logistic regression and used as the measure of effect, the odds ratio (OR).

In addition, we followed the proposal of Mishra et al. (2008) and test the variables alcohol, tobacco, and work, have effects of accumulation, critical period or mobility on academic progress (Supplementary file: Table S1 and Table S2 shows the specifications and constraints for the tests and the definitions of each effect, respectively). The first hypothesis, accumulation, assumes that lifetime exposure time affects the outcome. The critical period hypothesis considers that there is a certain period of life that has the highest influence on the outcome. The mobility hypothesis considers the status changes between different categories of a variable. This hypothesis assumes that these changes of position over life course explain the outcome.

## Results

Characteristics of the 1993 birth cohort population are shown in Table 1. Of the 4,104 respondents aged 18, 3,852 provided information on their level of education, 61% presented an age-grade gap. Most of the adolescents in the sample were white (63.7%), had mothers and fathers with five to eight years of schooling (49% and 50.6%), and families with four or five members (55.3%). The great majority of those interviewed did not report working at ages of 11 and 15 (95.7% and 81.5%, respectively). Regarding risky behaviours, most adolescents gave a negative answer to tobacco and alcohol use (77% of the adolescents have never started smoking).

**Table 1.** Descriptive statistics of the sample of the 1993 birth cohort in Pelotas, RS, Brazil.

Variable	N	%	95% IC	% age-grade gap at age 18
Total	3,852ª	100		61.0
Gender				
Female	1,952	50.67	49.1–52.2	53.38
Male	1,900	49.33	47.7–50.9	68.89
Skin color				
White	2,351	63.68	62.1–65.2	53.93
Non-white	1,341	36.32	34.8–37.9	73.68
Maternal educational level (years)				
≥12	257	6.68	5.9–7.5	16.34
9–11	1,052	27.34	25.9–28.7	54.66
5–8	1,886	49.01	47.4–50.6	64.63
0–4	653	16.97	15.8–18.2	78.56
Paternal educational level (years)				
≥ 12	215	6.0	5.2–6.8	15.35
9–11	996	27.77	26.3–29.2	52.31
5–8	1,817	50.67	49.0–52.3	64.72
0–4	558	15.56	14.4–16.7	74.37
Family size (people)				
2–3	774	20.83	19.5–22.1	53.36
4–5	2,056	55.33	53.7–56.9	57.59
≥6	886	23.84	22.5–25.2	76.86
Socioeconomic level (quintiles)				
5º highest	675	18.68	17.4–20.0	24.44
4º	760	21.04	19.7–22.4	51.71
3º	744	20.59	19.3–22.0	63.84
2º	733	20.29	19.0–21.6	75.85
1º lowest	701	19.40	18.1–20.7	85.59
Working at age 11				
No	3,563	95.75	95.0–96.3	60.60
Yes	158	4.25	3.6–4.9	75.95
Working at age 15				
No	3,012	81.54	80.2–82.7	56.71
Yes	682	18.46	17.2–19.7	80.65
Working at age 18				
No	552	18.29	16.9–19.7	64.13
Yes	2,466	81.71	80.3–83.0	66.18
Alcohol mobility (11–15)				
No	3,258	94.85	94.0–95.5	59.58
Yes	177	5.15	4.4–5.9	73.45
Alcohol mobility (15–18)				
No	1,837	53.48	51.8–55.1	59.55
Yes	1,598	46.52	44.8–48.2	61.14
Started smoking (age)				
Never	2,974	77.21	75.8–78.5	54.61
≤12 years old	121	3.14	2.6–3.7	90.08
13–15 years old	433	11.24	10.3–12.3	82.68
>15 years old	324	8.41	7.6–9.3	80.25

ª Of the 4,104 cohort members in 2011, we registered the schooling of 3,852 participants.

Table 2 shows the analysis performed to test the hypotheses of risk accumulation, critical period, and social mobility for alcohol, tobacco and work, respectively. For alcohol, the mobility was the best-fit model with p = 0.8491 for the relaxed model. For tobacco use, the best-fits were the accumulation models, with p = 0.3249 and = 0.5832 for the strict and relaxed accumulation models, respectively. For work, the results showed best-fit for relaxed accumulation.

**Table 2.** Series of full and partial F-Tests for different hypotheses (adapted from Mishra et al. (2008)).

	Alcohol			Tobacco			Work		
Hypothesis	df	F-statistic	p-value	df	F-statistic	p-value	df	F-statistic	p-value
**Accumulation**									
Strict	6, 3435	13.99	0.0297	6, 3480	6.96	**0.3249**	6, 2836	34.94	<0.0001
Relaxed	4, 3435	9.77	0.0445	4, 3480	2.85	**0.5832**	4, 2836	4.82	**0.3066**
**Critical period**									
11 years	6, 3435	16.60	0.0109	6, 3480	171.29	<0.0001	6, 2836	76.26	<0.0001
15 years	6, 3435	13.57	0.0349	6, 3480	144.52	<0.0001	6, 2836	10.59	**0.1019**
18 years	6, 3435	17.52	0.0076	6, 3480	14.36	0.0259	6, 2836	84.77	<0.0001
**Mobility**									
11–15 years	5, 3435	9.16	**0.1028**	5, 3480	145.54	<0.0001	5, 2836	20.08	0.0012
15–18 years	5, 3435	6.26	**0.2818**	5, 3480	50.95	<0.0001	5, 2836	54.78	<0.0001
11–18 years	5, 3435	16.38	0.0058	5, 3480	14.79	0.0113	5, 2836	86.51	<0.0001
Relaxed	5, 3435	4.35	**0.4996**	5, 3480	171.10	<0.0001	5, 2836	72.20	<0.0001
**No Effect**	7, 3435	19.78	0.0061	7, 3480	177.79	<0.0001	7, 2836	86.56	<0.0001

The variables, alcohol, tobacco and work, were included in the adjusted logit model according to the best-fit of the accumulation tests, critical period, and mobility. Results showed that work and tobacco variables have an accumulation effect, whereas the alcohol consumption has a mobility effect. Thus, we include the work variable for each follow-up and analyze the tobacco variable according to the age when adolescents started smoking. To analyze the alcohol we included two dummies variables that analyze the transition from ages 11 to 15 and from ages 15 to 18 (1 = changed the status of consumption; 0 = if the consumption status was maintained in the period).

Table 3 shows the crude and adjusted associations between the academic progress and the independent variables, for the year 2011, when the participants were, on average, 18 years old. In the crude analysis, only alcohol mobility (15–18) was not associated with academic progress (p < 0.05), the other variables were statistically significant and their effects had the expected direction. In the adjusted analysis, working at ages 11 and 18 lost significance.

**Table 3.** The odds ratio of age-grade gap according to demographic, socio-economic and behavioural variables.

	Crude Analysis			Adjusted Analysis		
Variables	OR	95% IC	p	OR	95% IC	p
Gender			<0.001			<0.001
Female	1			1		
Male	1.93	(1.69–2.20)		2.54	(2.07–3.10)	
Skin Color			<0.001			<0.001
White	1			1		
Non-white	2.39	(2.06–2.76)		1.47	(1.19–1.82)	
Maternal educational level (years)			<0.001			<0.001
≥12	1			1		
9–11	6.17	(4.34–8.77)		2.60	(1.47–4.61)	
5–8	9.35	(6.63–13.19)		3.02	(1.71–5.32)	
0–4	18.75	(12.83–27.42)		3.55	(1.91–6.56)	
Paternal educational level (years)			<0.001			<0.001
≥12	1			1		
9–11	6.05	(4.09–8.94)		2.32	(1.27–4.25)	
5–8	10.12	(6.90–12.84)		2.93	(1.62–5.32)	
0–4	16.00	(10.55–24.28)		3.34	(1.77–6.32)	
Family size (people)			<0.001			<0.001
2–3	1			1		
4–5	1.18	(1.00–1.40)		1.44	(1.13–1.84)	
≥6	2.90	(2.35–3.58)		2.49	(1.84–3.38)	
Socioeconomic level (quintiles)			<0.001			<0.001
5º highest	1			1		
4º	3.31	(2.64–4.15)		2.36	(1.70–3.27)	
3º	5.45	(4.33–6.87)		3.27	(2.34–4.56)	
2º	9.71	(7.61–12.39)		5.11	(3.60–7.25)	
1º lowest	18.36	(13.95–24.15)		9.05	(6.13–13.37)	
Working at age 11			<0.001			0.062
No	1			1		
Yes	2.05	(1.41–2.97)		1.64	(0.97–2.77)	
Working at age 15			<0.001			<0.001
No	1			1		
Yes	3.18	(2.59–3.89)		1.66	(1.28–2.16)	
Working at age 18			<0.001			0.181
No	1			1		
Yes	1.09	(0.90–1.32)		0.84	(0.66–1.08)	
Alcohol mobility (11–15)			<0.001			0.006
No	1			1		
Yes	1.87	(1.33–2.64)		1.99	(1.10–3.20)	
Alcohol mobility (15–18)			0.344			0.022
No	1			1		
Yes	1.07	(0.93–1.22)		1.26	(1.03–1.55)	
Started smoking (age)			<0.001			<0.001
Never	1			1		
≤12 years old	7.55	(4.14–13.76)		4.11	(1.95–8.69)	
13–15 years old	3.96	(3.06–5.14)		3.89	(2.72–5.57)	
>15 years old	3.37	(2.54–4.48)		2.77	(1.88–4.56)	

Males and non-whites presented higher odds of the age-grade gap. Males presented odds of age-grade gap 2.54 higher than females. Non-white students had a 1.47 increase in the odds of falling behind at school compared to white adolescents. The odds of age-grade gap decreased with higher maternal and paternal school levels. Having a mother with less than 5 years of schooling increases the odds of falling behind at school by 3.55, compared to having a mother with 12 or more years of schooling. The lower the family’s socio-economic level is, the higher the odds the student has of falling behind.

Bigger family size showed to be detrimental to academic progress; being a member of a family of six or more people increased the age-grade gap odds in 2.49, compared to small families (2–3 members). In relation to work, the fact of the adolescent having a job at age 15 represents an increase of 1.66 in the odds of falling behind at school.

Young people who engaged in risk behaviours – alcohol consumption, smoking – increase their odds of the age-grade gap. Start smoking before age 13 represents an increase in the odds of falling behind by 4.11 compared to adolescents who never started smoking. The sooner the adolescents start smoking the higher the odds of the age-grade gap. The alcohol consumption variables were entered into the model in accordance with the hypothesis of having a mobility effect. Thus, changing the status consumption from ages 11 to 15 represents an increase in the occurrence of an age-grade gap in comparison to those whose statuses remained unchanged. It is worth noting that among young people who have changed their consumption statuses between the ages from 11 to 15, about 83.6% of them started consuming alcohol, whereas only 16.4% of them stopped consuming. The same is true for alcohol mobility from ages 15 to 18 (Supplementary file: Table S3 and table S4, show the trajectory and the frequency of alcohol mobility variables).

## Discussion

This study sought to identify social and behavioural factors associated with the age-grade gap in a birth cohort in southern Brazil. We analyzed the educational results of the students at the ages of 18. The main socio-economic and demographic variables that had an association with falling behind at school were: gender, skin colour, schooling of the mother and the father, family size, and socio-economic level.

Males had a higher odds of the age-grade gap. The odds of higher academic failure on the part of the boys is reported in other studies (Machado & Gonzaga, 2007; Pontili & Kassouf, 2007), including cohort studies (Damiani, 2006; Vieira et al., 2012). One of the explanations would be that boys often help their parents at work, unlike what occurs with girls, who normally help with domestic chores (Machado & Gonzaga, 2007). Studies indicate that work undermines more intensely boys’ educational outcomes (Artes & Carvalho, 2010) and that domestic chores would not have a negative effect on education (IBGE, 2007). According to the International Labour Organization (ILO), 2.9 million Brazilian children and adolescents aged between 5 and 14 work outside their homes, and two-thirds of them are males (Vieira et al., 2012).

The higher odds of the age-grade gap in non-white adolescents have also been observed in other studies (Albernaz, Ferreira, & Franco, 2002; Machado, 2007; Machado & Gonzaga, 2007; Pontili & Kassouf, 2007; Vieira et al., 2012). Albernaz et al. (2002) and Andrade and Dachs (2007) highlight the inequality of opportunities in Brazilian schools, noting poorer results for non-white students of all socio-economic levels. A possible explanation would the existence of prejudice and discrimination racial in schools. Teachers have lower expectations regarding the performance of black students (Silva, Barros, Halpern, & Silva, 1997). Discrimination, expressed in different ways, on the part of teachers affects the individual attention received by students. Other evidence links the very aspiration of the student to the poor educational outcome. Black students would have lower aspirations in terms of performance, due to the fact their future expectations tend to be constantly thwarted in a discriminatory sociocultural context (Silva et al., 1997).

Paternal and maternal schooling have a fundamental role in educational results. The lower educational level of the parents increases the odds of the age-grade gap in the children.’ Several works report a positive association of paternal (Machado & Gonzaga, 2007) and maternal (Machado, 2007; Machado & Gonzaga, 2007; Menezes-Filho, 2007; Vieira et al., 2012) schooling in the educational achievements of the children.

Family size showed to influence the educational outcome. Children belonging to larger families have higher odds of presenting failure in academic progress. Competition for materials and household resources is more intense in larger families. The hypotheses of dilution of resources and sibling rivalry are plausible explanations for the existing negative relationship between family size and educational outcomes (Marteleto, 2002). A similar result was found by Menezes-Filho (2007).

Students of families with a lower socio-economic level have a higher odds of falling behind, corroborating literature (Albernaz et al., 2002; Machado & Gonzaga, 2007; Pontili & Kassouf, 2007; Vieira et al., 2012). Families with higher financial resources normally occupy a cultural and socio-economic context that is favourable to the build-up of human capital. A higher income also allows for the acquisition of goods – books, school materials, Internet access – that facilitate learning (Machado, 2007).

Adolescence is considered a transition stage in which concerns to behaviour and health-care gain strength. The development of the brain and puberty brings a new set of capabilities leading to new behaviours and different lifestyles (Viner et al., 2012). Certain habits acquired during this stage can be detrimental to health and to academic performance, undermining prospects during adulthood (Koivusilta, Arja, & Andres, 2003).

In adolescence, many young people enter the labour market. The need to generate an income and the search for financial independence are factors which lead to early working. The present study indicates that entering the labour market increases the odds of falling behind 66%, compared to adolescents who do not work at age 15. The same impact of work was found by Menezes-Filho (2007).

Behaviours acquired during adolescence tend to prevail in adulthood. Habits such as consuming alcohol and smoking bring short and long-term health risks (Ferreira, Torgal, & Reis, 2010). According to our results, participants who consumed alcohol or used tobacco had greater odds of falling behind. Our results for tobacco and alcohol are consistent with the findings of earlier studies (DeBerard, Spielmans, & Julka, 2004; Koivusilta et al., 2003).

The results here suggest that socio-economic factors and behavioural characteristics are associated with educational performance. Risk behaviours appear to exert a strong influence on academic performance. In adulthood, the cessation of certain acquired habits is difficult, and therefore, certain behaviours adopted in adolescence deserve the attention of the government.

The school, as an educational institution, can help young people overcome many cultural and historical barriers linked to skin colour/race that affect health behaviours at other times in life. Public policies that seek to provide education should be targeted at the most vulnerable groups, decreasing the observed inequalities. Awareness policies should target parents and students with information about future returns on educational investment, seeking to decrease the percentage of young people of school age in the labour market. Policies that aim at inhibiting certain risk behaviours also seem to be fundamental, broadly benefiting the population.

Our work has limitations by not considering other possible determinants of failure in academic progress. Ability, mental health conditions, characteristics referring to the parents’ preferences, and regional aspects such as the offer of schools and resources can influence the academic performance. However, they were not taken into account due to restrictions of the database that was used. The literature on the subject is still limited, especially in Brazil, and studies with longitudinal data are scarce. Nevertheless, our research is an important contribution to the literature.
