# Supplementary material for: Association of risk behaviours, socio-economic characteristics and academic progress in adolescents: an analysis of the 1993 birth cohort in Pelotas, Brazil
Source: Int J Adolesc Youth. 2019 Jan 11;24(4):474–83. doi: 10.1080/02673843.2018.1564932 (PMC6817324; doi:10.1080/02673843.2018.1564932)
Supplement: Supplemental Material [file RADY_A_1564932_SM8625.docx]

**SUPPLEMENTARY FILE**

Table S1 – Models specifications

| Model | Specifications | Constraints |
| --- | --- | --- |
| **Accumulation** |  |  |
| Strict | $\alpha+\beta_{1}+\beta_{2}+\beta_{3}$ | $\beta_{1}=\beta_{2}=\beta_{3};$ $\beta_{12}=\beta_{23}=\beta_{13}= \beta_{123}$ = 0 |
| Relaxed | $\alpha+\beta_{1}+\beta_{2}+\beta_{3}$ | $\beta_{12}=\beta_{23}=\beta_{13}= \beta_{123}$ = 0 |
| **Critical period** |  |  |
| 11 years | $\alpha+\beta_{1}$ | $\beta_{2}= \beta_{3}= \beta_{12}=\beta_{23}=\beta_{13}= \beta_{123}$ = 0 |
| 15 years | $\alpha+\beta_{2}$ | $\beta_{1}= \beta_{3}= \beta_{12}=\beta_{23}=\beta_{13}= \beta_{123}$ = 0 |
| 18 years | $\alpha+\beta_{3}$ | $\beta_{1}= \beta_{2}= \beta_{12}=\beta_{23}=\beta_{13}= \beta_{123}$ = 0 |
| **Mobility** |  |  |
| 11-15 years | $\alpha+\beta_{1}+\beta_{2}+\beta_{12}$ | $\beta_{12}=-(\beta_{1}+\beta_{2});$ $\beta_{3}=\beta_{23}=\beta_{13}= \beta_{123}$ = 0 |
| 15-18 years | $\alpha+\beta_{2}+\beta_{3}+\beta_{23}$ | $\beta_{23}=-(\beta_{2}+\beta_{3});$ $\beta_{1}=\beta_{12}=\beta_{13}= \beta_{123}$ = 0 |
| 11-18 years | $\alpha+\beta_{1}+\beta_{3}+\beta_{13}$ | $\beta_{13}=-(\beta_{1}+\beta_{3});$ $\beta_{2}=\beta_{12}=\beta_{23}= \beta_{123}$ = 0 |
| Relaxed | $\alpha+\beta_{1}+\beta_{2}+\beta_{3}+\beta_{12}+\beta_{23}$ | ${\beta_{23}=-(\beta_{2}+\beta_{3}); \beta}_{12}=\beta_{23}=-\beta_{2};$ $\beta_{13}= \beta_{123}$ = 0 |
| **No Effect** | $\alpha$ | $\beta_{1}=\beta_{2}= \beta_{3}=\beta_{12}=\beta_{23}=\beta_{13}= \beta_{123}=0$ |

Table S2 - Definitions for each hypothesis

| Hypothesis | Definition |
| --- | --- |
| **Accumulation** | **Longer-term exposure to variable (works, alcohol or tobacco) results in a proportionate increase the age-grade gap.** |
| Strict | Regardless of life-stage, the more occasions a respondent have the variable (works, alcohol or tobacco) the greater the effect on academic progress. This is modelled by constraining the effect size to be equal across all three life-stages. |
| Relaxed | Variable (works, alcohol or tobacco) at each time-point contributes to the increase in the age-grade gap, but that these effects do not have to be equal (i.e. can have differing effect sizes in the association with academic progress). |
| **Critical period** | **Assumes that variable (works, alcohol or tobacco) at only a specific life-stage will be associated with academic progress.** |
| 11 years | 11 years old represents a critical period, others periods have no effect. |
| 15 years | 15 years old represents a critical period, others periods have no effect. |
| 18 years | 18 years old represents a critical period, others periods have no effect. |
| **Mobility** | **Upward mobility has a negative impact on academic progress.** |
| 11-15 years | Mobility from ages 11 to 15. |
| 15-18 years | Mobility from ages 15 to 18. |
| 11-18 years | Mobility from ages 11 to 18. |
| Relaxed | Mobilities at any life-stage. |
| **No Effect** | **Assumes that variable (works, alcohol or tobacco) has no association with academic progress. Modelled by removing the variable from the regression models.** |

Table S3 -Trajectory and frequency for alcohol mobility (11-15) (n =3,435)

| Trajectory | | Frequency | |
| --- | --- | --- | --- |
| 11 | 15 | n | % |
| 0 | 0 | 3,252 | 94.67 |
| 0 | 1 | 148 | 4.31 |
| 1 | 0 | 29 | 0.84 |
| 1 | 1 | 6 | 0.17 |

Table S4 - Trajectory and frequency for alcohol mobility (15-18) (n = 3,435)

| Trajectory | | Frequency | |
| --- | --- | --- | --- |
| 15 | 18 | n | % |
| 0 | 0 | 1,683 | 49.00 |
| 0 | 1 | 1,598 | 46.52 |
| 1 | 0 | 47 | 1.37 |
| 1 | 1 | 107 | 3.11 |
